# The Effect of a Restraint Reduction Program on Physical Restraint Rates in Rehabilitation Settings in Hong Kong

**DOI:** 10.1155/2011/284604

**Published:** 2011-09-06

**Authors:** Claudia K. Y. Lai, Susan K. Y. Chow, Lorna K. P. Suen, Ivan Y. C. Wong

**Affiliations:** ^1^School of Nursing, The Hong Kong Polytechnic University, Hong Kong; ^2^The Nethersole School of Nursing, The Chinese University of Hong Kong, Hong Kong

## Abstract

*Background*. In view of the adverse effects of using restraints, studies examining the use of restraint reduction programs (RRPs) are needed. *Objectives*. To investigate the effect of an RRP on the reduction of physical restraint rates in rehabilitation hospitals. *Methods*. A prospective quasi-experimental clinical trial was conducted. Demographic data, medical and health-related information on recruited patients from two rehabilitation hospitals, as well as facility data on restraint rates were collected. *Results*. The increase in the restraint rate in the control site was 4.3 times greater than that in the intervention site. Changes in the restraint mode, from continuous to intermittent, and the type of restraint used were found between the pre- and postintervention periods in both the control site and the intervention site. *Discussion*. Compared with that in the control site, the RRP in the intervention site helped arrest any increase in the restraint rate although it had no effect on physical restraint reduction. The shift of restraint mode from continuous to intermittent in the intervention site was one of the positive outcomes of the RRP.

## 1. Introduction

Restraint is defined as any device, material, or equipment attached to or near a person's body that cannot be controlled nor easily removed by the person, and that deliberately prevents or is deliberately intended to prevent a person's free body movement to a position of choice and/or a person's normal access to his body [1]. Physical restraints may include vests, straps, wrist ties, splints, mitts, belts, geri chairs, and bed-side rails [2]. The prevalence of physical restraint varies from 5 to 56% as reported in existing literature. Two decades ago, in long-term care facilities in the United States, the prevalence of physical restraints was reported to be 28 to 37% [3]. Following the imposition of stricter regulations, the prevalence fell to about 5% in 2007 [4]. In some European nursing homes, however, prevalence rates are still high at 26 to 56% [5, 6]. A recent study has reported that the prevalence of physical restraint use in nursing homes varies more than five-fold across different countries, from an average of 6% in Switzerland to 9% in the US, 20% in Hong Kong, 28% in Finland, and over 31% in Canada [7]. Although Hong Kong does not have the highest restraint rate among the countries covered by the study, it still has a much higher restraint rate than Switzerland or the US. According to a local study, 75% of nursing staff in Hong Kong indicated that they had used different degrees of physical restraint in the previous three months of the study period [8].

The problem with the use of physical restraints is not limited to the ethical dilemma of impinging on a person's autonomy. Previous studies show that the use of physical restraints is associated with an increased risk of mortality [9–11] and morbidity, such as due to increased instances of falling [12], greater cognitive decline, increases in the occurrences of nonsocial behavior, increases in bladder and bowel incontinence, increases in new pressure sores and nosocomial infections, a rise in dependency for undergoing activities of daily living and walking, and increases in agitation [13]. 

Despite numerous reports on the side effects of physical restraints, unfortunately, only a few local scholars and health professionals have paid attention to the phenomenon of restraint use. Besides studies showing the relatively high prevalence rate of restraint use in local nursing homes and long-term care facilities, clinical observations by many health professionals also attest to the fact that the use of physical restraints is widespread. To ensure good quality care, the issue of restraint use, therefore, deserves better attention.

As mentioned by many healthcare workers, the major justifications for the use of physical restraints are patient-oriented. They include the maintenance of patients' safety, management of their agitation and aggression, control of their behavior, preventing patients from wandering, and extension of physical support. However, as observed in many nursing homes or hospitals, physical restraints may also be used for the convenience of healthcare workers, for the attainment of organizational goals (such as to complete work schedules), to maintain a comfortable social environment (such as by stopping residents from bothering others), and to facilitate treatment (such as by preventing patients from tampering with medical devices or from removing clothing or dressings and catheters) [14].

Though restraint rates were high prior to intervention, research findings show that reducing rates of restraint to 5% or below is achievable [15]. In a comprehensive review by Guttman and colleagues, several prospective randomized trials were noted to have attained impressive outcomes, such as reducing the prevalence of restraint use by 20% [15]. Previous studies demonstrate that carefully orchestrated programming can greatly reduce the use of physical restraining devices [16–19]. 

Education programs, consisting of a full-day seminar and a one-hour guidance session per month over six months, focused on the decision-making process in the use of restraints and alternatives to restraints consistent with professional practice and quality care, can reduce the number of restraint cases by 54% [20]. In addition, a six-month educational program combined with unit-based, resident-centered consultation can effectively and safely reduce the use of physical restraint in nursing homes by 56% [21].

As a summary of the overseas studies mentioned above, a reduction of the prevalence rate of physical restraint use to 5% or lower is achievable through implementation of well-structured programs that involve both education and advance administration-committee consultation. However, to what extent the same could be applied to local settings is still unknown. This study is a prospective quasi-experimental clinical trial aimed at reducing the use of physical restraint on patients in rehabilitation settings. Some local studies that explore the physical restraint rate, knowledge, and practice of nurses can be found. In Hong Kong, the rate of bedrail use was about 62.5% in local nursing homes, while the rate of use of other physical restraints was about 25% [22]. Another local study mentioned that 69% of nursing staff reported that they had used at least one form of physical restraint in the previous three months [23]. The paucity of local interest illustrates the immediacy of the need for attention to restraint education for health professionals and other health workers. Through the investigation of this important yet neglected phenomenon, health-service providers would be able to reflect on their manner of delivery of health services.

## 2. Methods

### 2.1. Setting and Sampling

This project was a collaborative effort between two rehabilitation hospitals and a school of nursing (SN). Two rehabilitation centers serving fairly similar patient groups were recruited into the study. One was designated as the intervention site (hereafter refer to as the study site for ease of discussion), while the other served as the control site. The adult psychiatry units in the control site were excluded because restraint use in these units was mainly for the management of possibly violent behaviors. There were no other exclusion criteria during the selection of subjects, as this study aimed to be a facility-wide endeavor for a change of practice. All patients in both settings were approached and invited to join the study. 

Approval for the study was obtained from the ethics committee of both rehabilitation hospitals and the university. Informed written consent was obtained from the patients or their proxies prior to the collection of data. 

### 2.2. Intervention

The study site underwent the restraint reduction program, while the control site did not. The restraint reduction program consisted of two components—staff education and the setup of a restraint reduction committee (RRC) and was implemented after a period of baseline observation. 

#### 2.2.1. Staff Education

All staff members were educated using the staff education package (both in Chinese and in English) developed based on (i) literature search, (ii) the research team's clinical experiences, and (iii) our analysis of the pre-intervention staff attitudes, beliefs, and behavior on the use of restraints. The content of the education package include is myths and misconceptions about restraint use, the facts and evidence of restraint use, what is restraint and understanding why we are using them, how to deal with the fear of patients' falls, are restraint alternatives available, caring for special patient populations and preventing them from being restrained. Discussions, simulation exercises, and case studies were used as much as possible to render the content more relevant to real life practice.

Similar contents, but with different levels of detail, were provided for health professionals and unregulated healthcare workers. The period of staff education lasted one year, from October 2002 to November 2003. Twelve repeating one-hour sessions were conducted. Members of the team attended staff meetings, case conferences, and unit meetings to facilitate the mastery of knowledge and skills development of the participants in the workplace. Staff could consult with the project team for uncertainties and on an individual basis during, for instance, RRC meetings, ward rounds, and personal encounters between the staff and members of the project team.

#### 2.2.2. Restraint Reduction Committee

After the staff education program was completed, the RRC meetings commenced. The RRC was formed by an interdisciplinary committee, whose members included doctors, nurses, occupational therapists, physical therapists, and social workers. It was formed with the aim of changing the behaviors of nursing staff. The existing physical restraint policy in the rehabilitation center was compared with that proposed in the literature. We found that they were similar, and no revisions were needed before the study could begin. In fact, both facilities are under the Hospital Authority of Hong Kong (a government-funded independent body) and are expected to comply with the Hospital Authority's guidelines in the use of physical restraints. A nurse specialist in the center's RRC provided consultation to staff, and the team conducted further in-service sessions for reinforcement or for the orientation of new staff. Alternatives to the use of restraints were introduced to the staff during this period. Patients who were restrained were reviewed by the RRC. The RRC meeting was incorporated into the weekly case conferences in the medical and geriatric units. The multidisciplinary team would identify the circumstances leading to the use of restraints, the presence and the feasibility of alternatives to restraints, and possible solutions during the case conferences. 

The same protocol was used in both hospitals during data collection. Subject data of individual patients were collected when they gave their consent to participate. Subjects who were restrained were reviewed by the RRC during the case meeting at the study site. 

### 2.3. Data Collection

There was a monthly baseline observation for five months in all centers. The outcome variables (restraint, risk of fall, and functional level) were collected (before intervention). After obtaining the baseline data, there was a two-month period of staff education. Each staff education period lasted for one hour, and 12 sessions were given during the two-month period. After the conduct of staff education, the RRC reviewed the cases of restraint for another four months. Data were again collected (after intervention). This post intervention data-collection period lasted for five months.

The prevalence of restraint was calculated as the “number of patient-restraint days/total number of patient days ×100”. The patient-restraint day is equal to one patient being restrained for any amount of time within a 24-hour period. 

Since most patients would be discharged before the end of the study, the mean scores on key variables (e.g., restraint, risk of fall, and functional level) were used as the unit of analysis. Data on the clinical and demographic characteristics of patients who were and those who were not being restrained in both the pre- and postintervention periods and the prevalence of restraint use were obtained.

All data were collected by research assistants who were trained by members of the team. Use of staff in the facilities could introduce bias. Training was provided for all research assistants until at least the conventional interrater agreement of 0.8 or more could be achieved. Assessment tools (e.g., MMSE) were administered by the RAs, while other data (e.g., restrained status) were collected through direct observations and examination of charts. Staff and patient interviews were conducted if there were discrepancies in the data. 

### 2.4. Instruments

Demographic data and medical and health-related information were collected once subjects were enrolled in the study. These included gender, consumption of psychoactive drugs, vision and hearing ability, restraint status, type of restraint used, and fall experience in hospital. The Cantonese version of the Mini-Mental State Examination (MMSE), the Morse Fall Scale (MFS), and the Modified Barthel Index (MBI) were used to assess the cognitive status, risk of fall, and functional level of the subjects. 

The Cantonese version of the MMSE was validated by Chiu et al. and found to have a Cronbach's alpha of 0.86 [24]. In this version, 19/20 (out of a total score of 30) was found to be the appropriate cut-off point for cognitive impairment in the local population, with a reliability rate of 97.5% and a validity rate of 97.3%. The interrater correlation was 0.99. The MFS was validated in Chinese hospital populations by Chow et al. and had a sensitivity rate of 31% and a specificity rate of 83% when the cut-off point was determined at 45 [25]. The field test demonstrated excellent interrater reliability with an ICC value of 0.97 (95% CI 0.94–0.98) [25]. For the MBI, the test-retest reliability of the Chinese version at the item level was comparable with that of the original version, with kappa statistics ranging from 0.63 to 1.00 (*P* < 0.001). Factor analyses revealed a two-factor structure that explained 75.7% of the total variance. Factor 1 was found to consist of eight items relating to the functional performance of patients. Factor 2 consisted of the two items that focused on patients' physiological needs [26]. 

## 3. Results

The samples in the two hospitals were first compared in terms of their demographic and clinical characteristics. The preintervention sample at the intervention facility was considerably older—mean age 75.4 (SD 10.7) versus 59.1 (SD 17.4) of the control facility (*P* < 0.001). The two samples were different in terms of the number of medical diagnosis, with the study site having a mean of 3.1 diagnoses (SD 1.5) versus the control site's mean of 1.8 diagnoses (SD 1.2) (*P* < 0.001); the mean MMSE score of the study site was 16.7 (SD 7.0) versus control site's 25.4 (SD 5.3) (*P* < 0.001); the mean MBI score was 12.7 (SD 5.5) in the study site versus control site's mean 15.1 score (SD 3.2) (*P* < 0.001). There were no significant differences found in all the other clinical and demographic variables.

The postintervention sample at the study site remained considerably older, with a mean age of 74.8 (SD 10.6) versus 62.9 (SD 17.7) in the control site (*P* < 0.001). Again, the two samples were different in terms of the number of medical diagnosis, with the study site having a mean of 3.2 diagnoses (SD 1.5) versus control site's mean of 1.9 diagnoses (SD 1.2) (*P* < 0.001); the mean MMSE score of the study site was 16.4 (SD 7.2) versus control site's 24.4 (SD 6.5) (*P* < 0.001); the mean MBI score of the study site was 12.0 (SD 5.1) versus control site's mean 15.1 (SD 3.4) (*P* < 0.001). The two facilities were also significantly different in terms of their gender composition—49.0% males in the study site versus 34.8% males in the control site. No other significant differences were found in clinical and demographic variables between the two hospital samples in the post-intervention period.

Then the baseline sample profile of the patients before the intervention in the study and control site were compared (Tables 1(a) and 1(b)). The control site had a significantly younger patient group than the study site (*P* < 0.001) in the nonrestrained patient group. This younger patient group probably resulted in the higher cognitive status as reflected in the significantly higher mean MMSE total score (*P* < 0.001) and the lower risk of fall as reflected in the lower Morse Fall Scale score (*P* < 0.001) (Table 1(a)). Restrained patients had a similar sample profile in both the study and control sites. However, significant differences were found in age (*P* < 0.01) in both the nonrestrained and restrained groups of patients between the study and control sites. A significant difference in the experiences of falling in hospital (*P* < 0.01) was also found in the nonrestrained patients (Table 1(b)). 

Last, the profiles of those who were restrained and nonrestrained were compared in each of the study sites. Tables 2(a) and 2(b) provide information about the categorical variables of the samples recruited during the pre- and postintervention period in both sites. Significant differences were found in the MBI score (*P* = 0.016) and vision (*P* < 0.001) in the study site in the postintervention period when compared with the preintervention period. The mean MBI score was worse (30.3 versus 31.5) while there were more people with normal vision (39.2% versus 51.2%) after intervention.

For the control site, there were significant differences in the age (*P* = 0.039) and the Morse fall scale (*P* < 0.001) in the postintervention when compared with the pre-intervention phase. The mean age was older (59.1 versus 62.9) and the mean MFS score (20.7 versus 30.3) showed a slightly higher risk post-intervention.

To answer the primary research question of whether a reduction in restraint rates had been attained in the post-intervention period, the prevalence of restraint was calculated. In the control site, the overall restraint rate (including both intermittent and continuous) significantly increased from 2.6 to 8.3% (*P* < 0.000). Both intermittent and continuous use of restraint were significantly increased in the postintervention period, with intermittent restraint use increasing from 0.2 to 3.3% (*P* < 0.001) and continuous restraint use increasing from 2.4 to 5% (*P* < 0.001) (Table 3). 

In the study site, there was no significant difference in the overall restraint rate between the pre- and postintervention periods although the overall restraint rate had a slightly upward trend from 11.5% to 13.6% (*P* = 0.405) postintervention. There was a significant increase in the use of intermittent restraint, from 0.7 to 5.1% (*P* < 0.001), but a significant decrease of continuous restraint from 10.8 to 8.5% (*P* < 0.001) (Table 3) was also recorded.

We analyzed the subsample recruited into the RRC (those complex cases who were brought to the multidisciplinary team for discussion about their progress). In this subsample, we found that there was a significant increase of patients with their restraints taken off after RRC conference (0% versus 18.2%, *P* = 0.014). There was an insignificant but slight increase of intermittent restraint (from 14.5% to 18.2%, *P* = 0.317), and there was a significant decrease of continuous restraint after RRC conference (from 85.5% to 63.6%, *P* = 0.009). These data provided further evidence that there was a positive direction of changes in practice in the intervention site. There was a significant increase in the use of bedrails and lap tables and a significant decrease in the use of waist belts and jackets after intervention in the control site. The bed rail was the most common type of restraint (61.9%), followed by the jacket restraint and waist belt (both at 14.3%), with lap tables as the least common (9.5%) after intervention.

In the study site, there was a decrease in the use of lap tables (from 48.0% to 39.2%) after intervention, but at the same time, there was an increase in the use of bed rails (43.3% to 51.8%) at the study site (Table 2(b)). Bed rail was the most common type of restraint, followed by lap table (39.2%) and jacket restraint (8.6%) in the postintervention period.

Because any increase or decrease in physical restraint use may imply a concomitant change in the use of chemical restraint, the subjects' use of psychotropic medications were also examined. No significant differences were found in the use of psychotropic medications in the study site (*P* = 0.346) and the control site (*P* = 0.075) comparing pre- and postintervention data. Both sites had a low rate in the use of psychotropic drugs to begin with and remained as such after intervention. In the study site, only 3.3% of the subjects were prescribed psychotropic medications before intervention, and 2.3% were prescribed psychotropic drugs after intervention. In the control site, 2% of the subjects were on psychotropic medications, and none were prescribed (0%) after intervention. 

The staff profiles were examined to see if there might be the possibility of impacting on the study outcomes. In the intervention site, the staff response rate in the preintervention survey was 82.2% (*N* = 97) and 77.4% after intervention (*N* = 89). No significant differences were found in their gender (*P* = 0.535), years of clinical experience (*P* = 0.684), with or without on-the-job training about restraint use (*P* = 0.179) pre- and post-intervention. Also, no significant differences were found in terms of knowledge (*P* = 0.147), attitude (*P* = 0.071), and practice (*P* = 0.139) before and after intervention using Janelli et al.'s (1991) validated questionnaire. When further analysis were conducted, a subsample of nurses (of one unit) were found to have better knowledge after intervention (mean score results: 5.51 versus 6.23, *P* = 0.04).

In the control site, the staff response rate before intervention was 51.5% (*N* = 17) and 77.8% after intervention (*N* = 28). No significant differences were found in their gender (*P* = 0.431), years of clinical experience (*P* = 0.516), and with or without on the job training about restraint use (*P* = 0.948). Moreover, no significant differences were found in terms of knowledge (*P* = 0.436) and attitude (*P* = 0.498). There was, however, a significant decrease in their practice score (from 38.41 to 36.43, *P* = 0.048). The problematic practice behaviors included item C7 (I tell family members why the patient is being restrained), C8 (I explain to the patient why the restraint is being applied), and C9 (I tell the patient when the restraint will be removed). 

When comparing nursing staff samples of between the study and control sites, there were no significant differences observed in their gender distribution (before intervention: *P* = 0.279 and after intervention: *P* = 0.348), years of clinical experience (before intervention: *P* = 0.369; after intervention: *P* = 0.055), and with or without on the job training about restraint use (before intervention: *P* = 0.153; after intervention: *P* = 0.176). No significant differences were found in terms of knowledge (*P* = 0.230) and attitude (*P* = 0.986). However, there was a significant difference in their mean practice score (38.41 versus 36.43, *P* = 0.037), with the control site having relatively poorer scores.

## 4. Discussion

This prospective trial did not find a lowering of restraint rates with the implementation of a restraint reduction program. In contrary, findings from similar studies in other countries reported significant reductions in the use of restraints after the implementation of restraint reduction programs [20, 27, 28]. In the study site, an increase in the use of intermittent restraint and a concomitant decrease in the use of continuous restraint could mean that members of the staff were adopting a less restrictive policy. On the other hand, the control site had a significant increase in restraint rates despite there were almost no significant differences between the pre- and postintervention samples in terms of their clinical and demographic variables. There were significant differences in only two variables amongst many—age and MFS score—and the differences probably had limited clinical significance. The mean age of the control site sample after intervention was 2.8 years older than that of the preintervention sample, and a mean MFS score of 30 postintervention means a low risk of falls according to the psychometric properties of the MFS. Therefore, it is reasonable to conclude that although the overall restraint rate was not reduced in the study site, the interventions had certain positive outcomes. 

In Werner and Cohen-Mansfield's study, participants did not change their perceptions about restraint use over time despite the institution tried their best to prepare and informed them about changes in the restraint policy [29]. To bring about practice behavior changes is a challenging task. It has been a topic of interest in management studies for decades. This may be one of the reasons why the control site had poorer performance than that study site.

No study on the long-term sustained effects of restraint reduction programs in health care settings can be located in the literature. In an intervention study for family caregivers, Mittleman et al. reported that caregivers in the enhanced treatment group had significantly fewer depressive symptoms that sustained for 3.1 years after the intervention than did the control subjects [30]. The enhanced treatment group had ongoing ad hoc counseling available to them. In the same rein, ongoing education and repeated reminder of hospital guidelines in the study site might have been useful in inhibiting a more liberal use of physical restraint.

Changes in the type of restraint used were found in the control site. However, the distribution of the percentages of types of restraint used remained stable throughout the pre- and post-intervention periods. There was a significant increase in the use of bed rails, while the use of other types of restraint increased at about the same rate as the overall increase in restraint use. There was an increase in the use of bed rails in the control site that can probably be explained by the fact that bed rails are not considered a restraint by Hong Kong nurses. This of course is controversial. In some countries, whether or not bed rails should be considered a restraint is also a debatable issue [31]. In some institutions, bed rails, besides serving as a restraint, also serve as a bed mobility or transfer aid; thus, side-rail reduction is not as simple as lowering the rail [32–34]. Since the types of restraint used in the study and the control site were different, comparisons of the changes in its use cannot be made without further study. Regarding the significant changes in the sample profile of patients, we believe that the differences were mainly due to changes in the sample profile resulting from the entry of new patients. There was a significant reduction in the MBI post-intervention in the study site compared with that in the control site. However, there was actually a 0.68-unit decrease in the mean MBI score. This level of decrease is not clinically significant when considering the functional mobility of a patient.

There was a significant increase in the mean MFS score in the control site postintervention compared with that of the preintervention period. The MFS is a widely used scale for assessing a patient's likelihood of falling [25, 35]. A higher score in the scale represents a higher risk. The change in the mean MFS score from 20.74 (±15.45) before intervention to 30.26 (±16.91) after intervention represented a change from no risk (0–24) to low risk (25–50) in the control group. Some may argue that the increase in the mean MFS score might lead to an increase in the use of restraints in the control site. According to previous studies, an increase in the risk of falling [36–38] and an increase in the dependency of patients [37] are factors that could increase the restraint rate. However, the decrease in the MBI score in the study site did not result in an increase in the use of restraints. Similarly, a progression from a “no risk” to “low risk” in the risk of falling does not necessarily result in an increase in the restraint rate. Successful translation of research into clinical practice guidelines that improve health care is complex and requires a multifaceted strategy. To facilitate restraint reduction to a larger extent and on a more significant level, further studies on the components of a restraint reduction program are necessary. This may include the depth and duration of staff education, and more frequent multidisciplinary staff meetings between staff and the restraint reduction committee. 

## 5. Study Limitations and Suggestions for Further Studies

The data in this study were collected from a convenience sample recruited from only two rehabilitation hospitals in Hong Kong. Thus, the external validity of the findings is limited. The differences in various dimensions of the two rehabilitation hospitals might also lead to potential differences between the intervention and control samples. Moreover, personal influence from ward supervisors might have led to the contamination of results in the study site during the project, since management tended to interfere with the decision of the staff on whether to use restraints.

## 6. Conclusion

There is vast literature on the disadvantages of physical restraint use in health care settings. However, the use of physical restraints is still a common practice in Hong Kong. Based on the results of this study, the implementation of a restraint reduction program may be able to bring about certain positive outcomes. Sustained monitoring of restraint use through the work of an interdisciplinary restraint review committee and staff education may be able to help reduce restraint use. 

Researchers and clinicians in developed countries have been working on the issue of restraint reduction for two decades. Now we have some initial local evidence to show that, to a certain extent, restraint reduction efforts could be beneficial. The effects of the intervention program may be further investigated through the modification of both the educational program provided to healthcare workers and of the RRC. Modifications may include increases in the length of the education period, the depth of the educational content, and monitoring time by the RRC. We look forward to future reduction in the restraint rate in the local settings. A reduction in the restraint rate would unquestionably improve the quality of life of the patients under our care.

## Figures and Tables

**Table:** (a) Sample profile before intervention (I)

	Nonrestrained			Restrained		
Mean/SD	Study site (*n* = 270)	Control site (*n* = 142)	*P*-value*	Study site (*n* = 128)	Control site (*n* = 6)	*P*-value*
Age	74.18/10.88	54.69/17.36	<0.001	78.03/9.88	69.67/14.43	0.16
MMSE	18.64/6.45	25.66/4.96	<0.001	12.71/6.39	18.50/7.82	0.053
MBI	14.30/5.00	15.23/3.08	0.69	9.23/5.05	11.33/3.98	0.221
Morse Fall Scale	29.98/16.80	20.77/15.37	<0.001	31.05/17.85	20.00/18.71	0.085

MMSE: Mini-Mental State Examination; MBI: Morse Fall Scale.

**Table:** (b) Sample profile before intervention (II)

		Nonrestrained			Restrained		
	%	Study site (*n* = 270)	Control site (*n* = 142)	*P*-value*	Study site (*n* = 128)	Control site (*n* = 6)	*P*-value*

Gender	M	52.6	40.1	0.016	33.6	83.3	0.013
	F	47.4	59.9		66.4	16.7	
Psychoactive drug	Yes	1.9	2.1	0.855	6.3	0	0.528
	No	98.1	97.9		93.8	100	
Vision	Normal	39.6	43	0.514	38.3	33.3	0.807
	Impaired	60.4	57		61.7	66.7	
Hearing	Normal	84.1	88.7	0.2	80.8	83.3	0.862
	Impaired	15.9	11.3		19.2	16.7	
History of fall in hospital	Yes	99.3	95.1	0.006	100	100	N/A
	No	0.7	4.9		0	0	

**Table:** (a) Sample profile of study and control sites (I)

				Age	No. of diagnoses	MMSE	MBI	Morse fall scale
Intervention site	Preintervention phase	*n* = 398	Mean ± SD	75.42 ± 10.71	3.05 ± 1.45^a^	16.74 ± 7.00	12.67 ± 5.54	30.33 ± 17.13
			Median/range	77/31–100	3/1–7	17/0–30	13/0–20	25/0–80
	Postintervention phase	*n* = 612	Mean ± SD	74.80 ± 10.57	3.15 ± 1.50^b^	16.42 ± 7.15	11.99 ± 5.12	31.54 ± 17.13^a^
			Median/range	76/23–98	3/1–9	16/0–30	13/0–20	25/0–90
			P*	0.200	0.286	0.375	0.016	0.239
Control site	Preintervention phase	*n* = 148	Mean ± SD	59.14 ± 17.35	1.80 ± 1.159	25.37 ± 5.26	15.07 ± 3.20	20.74 ± 15.45
			Median/range	64/17–88	1/1–7	27/0–30	15/4–20	15/0–80
	Postintervention phase	*n* = 155	Mean ± SD	62.90 ± 17.73^c^	1.93 ± 1.21	24.39 ± 6.53	15.07 ± 3.41	30.26 ± 16.91
			Median/range	67/15–93	2/1–7	27/4–30	16/3–20	25/0–75
			P*	0.039	0.284	0.583	0.485	<.001

MMSE: Mini-Mental State Examination; MBI: Morse Fall Scale.

*Mann-Whitney *U* test, significant at 0.05.

^a^One missing subject.

^b^Three missing subjects.

^c^One missing subject.

**Table:** (b) Sample profile of study and control sites (II)

	Study site					Control site				
	Before intervention		After intervention		(*X^2^*)	Before intervention		After intervention		(*X^2^*)
	*n* = 398	%	*n* = 612	%	*P*-value	*n* = 148	%	*n* = 155	%	*P*-value

Gender										
M	185	46.5	300	49	0.43	62	41.9	54	34.8	0.207
F	213	53.5	312	51		86	58.1	101	65.2	
Psychoactive drug										
Yes	13	3.3	14	2.3	0.346	3	2	0	0	0.075
No	385	96.7	598	97.7		145	98	155	100	
Vision										
Normal	156	39.2	308	51. 2	<0.001	63	42.6	68	43.9	0.819
Impaired	242	60.8	293	48.8		85	57.4	87	56.1	
Hearing										
Normal	330	82.9	502	83.5	0.729	131	88.5	132	85.2	0.389
Impaired	68	17.1	99	16.5		17	11.5	23	14.8	
Restraint status										
Never^a^	270	67.8	309	50.5	<0.001	142	95.9	134	86.5	0.01
Intermittent^b^	36	9	147	24		4	2.7	9	5.8	
Continuous^c^	92	23.1	152	24.8		2	1.4	12	7.7	
Type of restraint										
Lap table	61	48	118	39.2	0.334	0	0	2	9.5	0.008
Vest	11	8.7	26	8.6		1	16.7	3	14.3	
Wrist	0	0	1	0.3		—	—	—	—	
Pelvic belt	—	—	—	—		2	33.3	0	0	
Waist belt	—	—	—	—		2	33.3	3	14.3	
Mittens	—	—	—	—		1	16.7	0	0	
Others (bed rails)	55	43.3	156	51.8		0	0	13	61.9	
History of fall in hospital										
Yes	2	0.5	6	1	0.402	7	4.7	10	6.5	0.515
No	396	99.5	606	99		141	95.3	145	93.5	

*P*-values: a versus b in Control site: <0.001, b versus c: <0.001, a versus c: <0.001.

*P*-values: a versus b in Study site: <0.001, b versus c: <0.001, a versus c: 0.013.

**Table 3:** Comparison of the pre- and postintervention restraint rates in the study and control sites.

	Study site			Control site		
	Preintervention phase	Postintervention phase		Preintervention phase	Postintervention phase	
	*n* = 463	*n* = 562	*P*-value (*t*-test)	*n* = 305	*n* = 310	*P*-value (*t*-test)
	M (%) ± SD	M (%) ± SD		M (%) ± SD	M (%) ± SD	

Intermittent restraint						
A/D	0.6 ± 0.02	7.1 ± 0.08	<0.001	0.4 ± 0.01	6.3 ± 0.05	<0.001
P/D	0.7 ± 0.02	3.7 ± 0.05	<0.001			
N	1.0 ± 0.02	2.8 ± 0.04	<0.001	0.0 ± 0.00	0.4 ± 0.02	0.019
Overall	0.7 ± 0.02	5.1 ± 0.07	<0.001	0.2 ± 0.01	3.3 ± 0.05	<0.001
Continuous restraint						
A	10.7 ± 0.12	8.3 ± 0.07	0.001	4.2 ± 0.03	6.6 ± 0.05	<0.001
P	11.6 ± 0.15	7.6 ± 0.07	<0.001			
N	10.0 ± 0.12	10.5 ± 0.07	0.592	0.6 ± 0.02	3.5 ± 0.03	<0.001
Overall	10.8 ± 0.13	8.5 ± 0.07	<0.001	2.4 ± 0.03	5.0 ± 0.05	<0.001

A/D: am shift for Study site; or day shift for Control site.

P/D: pm-shift for Study site; or day-shift for Control site.

N: night-shift.

*n*: number of shifts.

*restraint rate: the number of patients being restrained/the total number of patients in the ward per shift.
